# Rituximab for Children with Immune Thrombocytopenia: A Systematic Review

**DOI:** 10.1371/journal.pone.0036698

**Published:** 2012-05-30

**Authors:** Yi Liang, Lingli Zhang, Ju Gao, Die Hu, Yuan Ai

**Affiliations:** 1 Department of Pharmacy, West China Second University Hospital, Sichuan University, Chengdu, China; 2 West China School of Pharmacy, Sichuan University, Chengdu, China; 3 Department of Pediatric Hematology and Oncology, West China Second University Hospital, Sichuan University, Chengdu, China; Centre de Recherche Public de la Santé (CRP-Santé), Luxembourg

## Abstract

**Background:**

Rituximab has been widely used off-label as a second line treatment for children with immune thrombocytopenia (ITP). However, its role in the management of pediatric ITP requires clarification. To understand and interpret the available evidence, we conducted a systematic review to assess the efficacy and safety of rituximab for children with ITP.

**Methodology/Principal Findings:**

We searched MEDLINE, EMBASE, Cochrane Library, CBM, CNKI, abstract databases of American Society of Hematology, American Society of Clinical Oncology and Pediatric Academic Society. Clinical studies published in full text or abstract only in any language that met predefined inclusion criteria were eligible. Efficacy analysis was restricted to studies enrolling 5 or more patients. Safety was evaluated from all studies that reported data of toxicity. 14 studies (323 patients) were included for efficacy assessment in children with primary ITP. The pooled complete response (platelet count ≥100×10^9^/L) and response (platelet count ≥30×10^9^/L) rate after rituximab treatment were 39% (95% CI, 30% to 49%) and 68% (95%CI, 58% to 77%), respectively, with median response duration of 12.8 month. 4 studies (29 patients) were included for efficacy assessment in children with secondary ITP. 11 (64.7%) of 17 patients associated with Evans syndrome achieved response. All 6 patients with systemic lupus erythematosus associated ITP and all 6 patients with autoimmune lymphoproliferative syndrome associated ITP achieved response. 91 patients experienced 108 adverse events associated with rituximab, among that, 91 (84.3%) were mild to moderate, and no death was reported.

**Conclusions/Significance:**

Randomized controlled studies on effect of rituximab for children with ITP are urgently needed, although a series of uncontrolled studies found that rituximab resulted in a good platelet count response both in children with primary and children secondary ITP. Most adverse events associated with rituximab were mild to moderate, and no death was reported.

## Introduction

Immune thrombocytopenia (ITP) is an immune-mediated disease characterized by transient or persistent decrease of the platelet count and increased risk of bleeding [1]. The incidence of ITP is 1.9∼6.4 per 10^5^ children/year [2] and 23.1%∼47.3% of children with ITP suffer a disease course more than 6 months [3].

The goal of ITP treatment is to achieve a platelet count that is associated with adequate hemostasis, rather than a “normal” platelet count. The recommended first-line drug treatment for children with ITP includes corticosteroids, intravenous immunoglobulin (IVIg) and Anti-D immunoglobulin [4], but for pediatric patients refractory to first line treatment, appropriate second line treatments are needed.

Rituximab is a chimeric, monoclonal anti-CD20 antibody that targets B lymphocytes and causes Fc-mediated cell lysis [5], [6]. It was initially approved for the treatment of lymphoma, as it can substantially decrease the normal and malignant B-cells [7]. It has also been approved for the treatment of rheumatoid arthritis in Europe, as it can destroy B lymphocytes in the joints to help reducing inflammation [8]. In recent years, it has been widely used off-label as the second line treatment in both adults and children with ITP refractory to first line treatment. A systematic review based on studies on adult patients with ITP found that rituximab resulted in overall response (platelet count >50×10^9^/L) and complete response (platelet count >150×10^9^/L) in 62.5% and 43.6% of patients, respectively [9]. Several studies on children suggested that rituximab showed a similar effect as adult ITP, but the reported response rate varied among those studies [10]. In addition, those results may be potentially biased and imprecise, as most studies are case series, and only involved a relatively small number of patients. The role of rituximab in the management of pediatric ITP still requires clarification. To understand and interpret the available evidence, we conducted a systematic review to evaluate the efficacy and safety of rituximab for children with ITP.

## Methods

### Searching

We searched PUBMED, EMBASE, Cochrane Central Register of Controlled Trials (CENTRAL) published in Cochran Library (2012, Issue 1) using the search strategy detailed in table S1; we searched Chinese Biomedical Literature Database (CBM) and Chinese National Knowledge Infrastructure (CNKI) for literatures published in Chinese. We also searched the electronic databases of American Society of Hematology, American Society of Clinical Oncology and Abstract Database of Pediatric Academic Society including its Late-Breaker Abstract Presentations,with the search term ritux*, child* and pediatr*. The references of all retrieved articles were scanned for additional relevant citations. We searched all databases from their earliest records to January 2012.

### Eligibility Criteria

Clinical studies published in full text or abstract only were both included, and there was no restriction on study design or publication language. We included children aged less than 18 years with primary or secondary ITP, having platelet counts less than 30×10^9^ cell/L. Studies that included both children and adults were excluded if data of children could not be extracted separately. Patients were treated with rituximab irrespective of dosage and schedule. Outcome criteria based on guidance recently provided by international working group (IWG) consensus panel of both adult and pediatric experts [1]. Response (R) was defined as any platelet count ≥30×10^9^/L and at least doubling of the baseline count. Complete response (CR) was defined as any platelet count ≥100×10^9^/L. Time to response was from starting treatment to achievement of response. Duration of response was measured from the achievement to loss of response. All reported adverse events were included for safety assessment. Studies that enrolled fewer than 5 patients but failed to contribute to safety analysis were excluded.

### Study Selection and Data Abstraction

Two authors (Yi Liang and Die Hu) independently screened titles and abstracts of all studies identified by the search strategy and assessed the studies for inclusion using predetermined inclusion criteria. The full texts of all potentially relevant articles were retrieved for detailed review. We resolved disagreements by discussion until consensus is achieved.

Two authors (Yi Liang and Die Hu) used a pre-designed data collection form to independently extract data from each included study. The following data were extracted: (1) characteristics of patients, including number of patients that meet the inclusion criteria, country, age, primary/secondary ITP, duration of ITP, splenectomized or not, and platelet count before rituximab treatment; (2) study design and use of controls; (3) dose and schedule of rituximab; (4) number of patients with platelet count response, complete response, and their definitions; (5) time to platelet count responses; (6) follow-up and duration of platelet count responses; (7)toxicities associated with rituximab; (8) source of funding.

### Quality Assessment

Quality assessment was based on the checklist developed for assessment of case series by UK national institute for clinical excellence (NICE) [11]. We answered 8 questions included in the checklist with yes, no, or unclear. Quality assessment was only conducted in studies enrolling 5 or more patients, as those studies contributed to efficacy analysis.

### Statistical Synthesis

Platelet count responses were analyzed only from those studies enrolling 5 or more patients, as smaller studies may be subject to extreme risk of reporting and selection bias. Adverse effects were considered from all studies, including those enrolling fewer than 5 patients each. We achieved exact binomial 95% confidence interval (CI) of response rate reported in each study. We estimated the between-study variance and determined pooled estimates of response rate using the software STATA 11.1 with a random-effect model [12]. Time to response and response duration were described with medians, minimum and maximum values, and for studies that reported individual level data, we combined and summarized these data with medians and inter quartile ranges (IQR). We assessed publication bias both with Egger and Begg test in STATA 11.1, and P≦0.05 suggested a significant publication bias [13], [14].

We calculated a kappa statistic for measuring agreement between two authors making decisions on study selection. Values of kappa between 0.40 and 0.59 were considered to reflect fair agreement, between 0.60 and 0.74 to reflect good agreement and 0.75 or more to reflect excellent agreement [15]. The design and report of this review has been checked with PRISMA checklist (see table S2).

## Results

### Study Selection

**Figure 1:**
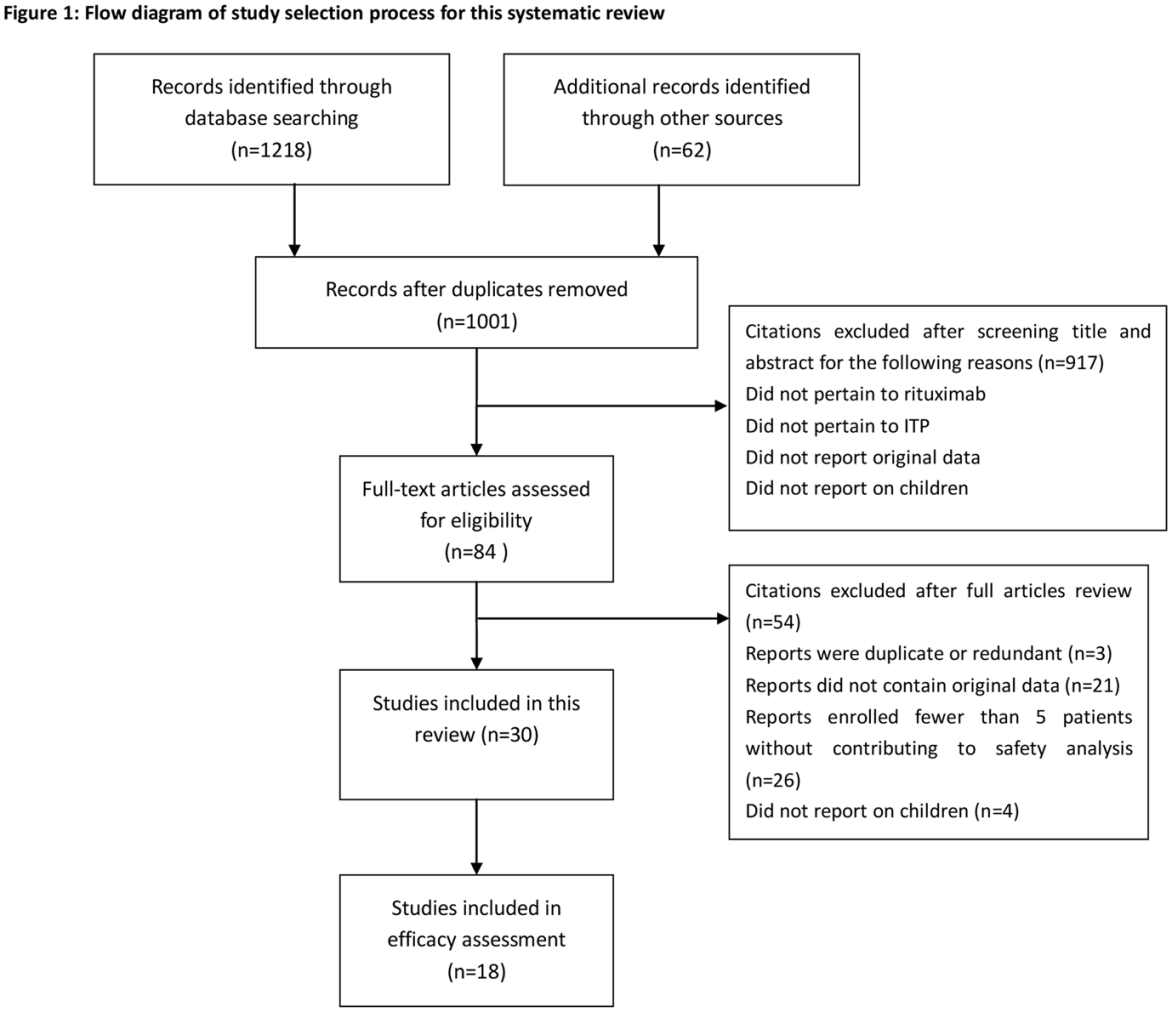
Flow diagram of study selection process for this systematic review. This is a modified four-phase PRISMA 2009 flow diagram that maps out the number of records identified, included and excluded, and the reasons for exclusions.

1280 citations were identified from the literature search, and 30 studies [16]–[45] (370 patients) were included in this systematic review. 18 studies [16]–[33] (352 patients) enrolled 5 or more patients each, among which 5 studies [29]–[33] (59 patients) were published only in abstract; 12 studies [34]–[45] (18 patients) enrolled less than 5 patients each, among which 2 studies [34], [38] (6 patients) were published in abstract. Figure 1 shows the literature selection process. Agreement between 2 reviewers for study selection was excellent (Κ = 0.75).

### Study Design and Source of Funding

17 case series [16]–[27], [29]–[33], 12 case reports [34]–[45] and 1 longitudinal observational cohort study were included in this systematic review. The cohort study included a control group involving patients without rituximab administration, but only reported concerned outcomes in the rituximab group, failing to make a comparison on effects between two groups [28]. 9 studies [17]–[19], [24], [28], [31], [37], [39], [40] were supported by non-profit organizations; 4 study did not receive any funding source [21], [22], [33], [42]; Source of funding was not reported in the other 17 studies.

### Quality Assessment

18 studies [16]–[33] enrolling 5 or more patients were involved in the quality assessment (table 1). 10/18 studies were multicenter studies; All studies described their hypothesis/objective and main findings clearly; 14/18 studies clearly defined outcomes; 9/18 studies clearly described patient inclusion criteria; 9/18 studies collected patients data prospectively, 1 cohort study collected data both prospectively and retrospectively; Only 1 study stated consecutive enrollment of patients, none of other studies provided this information, although it is necessary, as consecutive enrollment may reduce risk of selection bias; Platelet count response was stratified in 5 studies by clinical characters of patients to find predictors of response to rituximab.

**Table 1:** Quality assessment of 18 studies that contributed to efficacy analysis.

Study	Multi-center study	Objective clearly described	Inclusion/exclusion criteria clearly described	Clear definition of outcomes	Data collected prospectively	Patients recruited consecutively	Main finding clearly described	Outcomes stratified
Taube (2005) [16]	NR	Y	N	Y	Y	NR	Y	N
Wang (2005) [17]	Y	Y	Y	Y	Y	NR	Y	N
Bennett (2006) [18]	Y	Y	Y	Y	Y	NR	Y	Y
Bader-Meunier (2007) [19]	Y	Y	Y	Y	N	NR	Y	N
Rao (2008) [20]	Y	Y	Y	Y	Y	NR	Y	N
Dogan 2009 [21]	N	Y	N	Y	Y	NR	Y	N
Kumar (2009) [22]	N	Y	Y	Y	N	Y	Y	N
Parodi (2009) [23]	Y	Y	N	Y	Y	NR	Y	Y
Rao (2009) [24]	Y	Y	Y	N	N	NR	Y	N
Citak (2011) [25]	NR	Y	N	Y	Y	NR	Y	N
Xu (2011) [26]	Y	Y	Y	Y	NR	NR	Y	N
Yang (2011) [27]	N	Y	Y	Y	Y	NR	Y	N
Grace (2012) [28]	Y	Y	Y	Y	Y/N	NR	Y	Y
Sharma (2005)* [29]	NR	Y	NR	Y	NR	NR	Y	Y
Kim (2009)* [30]	NR	Y	NR	Y	NR	NR	Y	N
Pospisilova (2010)* [31]	Y	Y	NR	NR	N	NR	Y	N
Sampaio (2010)* [32]	Y	Y	NR	NR	N	NR	Y	N
Wang (2011)* [33]	N	Y	NR	NR	Y	NR	Y	Y

Y: yes; N: no; NR: not reported.

* Abstract.

### Description of Patients

352 patients were included in 18 studies enrolling 5 or more patients. Characteristics of these patients were described in table 2. 171 (48.6%) patients in 6 studies [17], [18], [20], [22], [24], [28] were from USA/Canada, and 181 (51.4%) patients were from other countries, including Germany [16], Italy [23], France [19] Czech Republic [31],Portugal [32], China [26], [27], [33], Turkey [21], [25], South Korea [30] and India [29]. 304 patients were diagnosed as primary ITP, and 48 cases were diagnosed as secondary ITP associated with other diseases, including Evans syndrome, systemic lupus erythematosus, and autoimmune lymphoproliferative syndrome. The patients were from 0.5 to 19 years old and had a duration of ITP from 0.2 to 175 months, with platelet count from 1 to 75×10^9^/L before rituximab treatment, according to studies that provided data on these items. Treatments before rituximab varied between and within studies. IVIG, steroid, and anti-D were most commonly used, other previous treatment included splenectomy, cyclophosphamide, ciclosporin, azathioprine, vincristine, danazol, hydroxychloroquine and mycophenolate mofetil.

**Table 2:** Characteristics of studies enrolling 5 or more patients each.

Study	Country	PrimaryITP, n	secondaryITP, n	Age, y	Splenectomized, n	ITP duration,mo	Platelet Countbefore rituximabtreatment, ×10^9^/L	Previoustreatment	Study design	Dosage ofrituximab,mg/m^2^/dose/week	Doses, n
Taube (2005) [16]	Germany	22	None	5.8 (2.5–15.2)Δ	2	44 (14–103)	5 (2–27)	IVIG, St, Anti-D, Sp	Case series	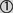	1
Wang (2005) [17]	USA	24	None	12.5 (2–19)	4	23 (6–120)	<30	Sp, IVIG, St, Anti-D, Dan, Az, VCR	Case series	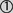	4
Bennett (2006) [18]	USA	30	6 (ES associated)	11.2 (2.6–18.3)	7	0.6–12.1	1–27	St,IVIG, Sp, Anti-D	Case series	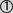	4
Bader-Meunier (2007) [19]	France	None	11 (ES associated)	7.7 (0.7–15)	6	1 (0.2–8.5)	NR	IVIG, CSA, Az, Cyc, Sp, VCR, Dan	Case series	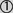	3–4
Rao (2008) [20]	USA	19	None	11 (3.8–18.6)	NR	1–48	NR	IVIG,St	Case series	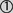 ; 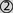	4–6
Dogan (2009) [21]	Turkey	10	None	3.3–13	0	NR	7 (2–20)	St, IVIG, anti-D	Case series	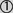	4–6
Kumar (2009) [22]	Canada	None	6 (SLE associated)	14 (8–16)	0	6(2–30)	31 (2–75)	St, IVIG, Az, HCQ	Case series	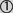 ; 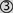	2–4
Parodi (2009) [23]	Italy	49	None	10.7 (1.2–17.7)	5	21(1–175)	7 (3–23)	IVIG, St,CSA, Sp	Case series	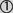	2–6
Rao (2009) [24]	USA	None	6 (ALPS associated)	10 (1–15)	5	NR	12 (2–16)	St, IVIG, Sp, MMF; VCR; Cyc;	Case series	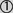	4
Citak (2011) [25]	Turkey	12	None	6 (4–14)Δ	NR	38 (14–98)	8 (2–28)	St, IVIG	Case series	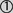	4
Xu (2011) [26]	China	9	None	6.8 (3–12)	2	16 (8–29)	14 (3–21)	St, Sp	Case series	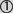	4
Yang (2011) [27]	China	9	None	6	NR	12–22	10–20	St, IVIG, VCR; CSA	Case series	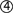	4
Grace (2012) [28]	USACanada	61	19#	7.5 (IQR 4.9, 12)Δ	NR	NR	14–40Δ	St, VIG	Longitudinal, observational, cohort	NR	NR
Sharma (2005)* [29]	India	10	None	9 (4–18)	6	NR	<10	St, CSA Cyc, VCR, IVIG Sp	Case series	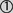	4
Kim (2009)* [30]	South Korea	11	None	6.5 (0.5–15.4)	1	NR	13.7 (3–46)	IVIG St Sp	Case series	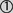	4
Pospisilova (2010)* [31]	Czech Republic	10	None	4–18	NR	NR	NR	St, IVIG, Az, anti-D	Case series	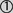	2–4
Sampaio (2010)* [32]	Portugal	7	None	4–18	1	27 (4–72)	NR	St, IVIG	Case series	NR	NR
Wang (2011)* [33]	China	21	None	NR	NR	NR	NR	NR	Case series	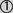 ; 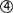	1–4

Results are given as median (range), unless otherwise noted.

* Abstracts only; NR: not reported; IQR: inter-quartile range.

Dosage of rituximab: ?375 mg/m^2^/dose/week; ?750 mg/m^2^/dose/week; ?500 mg/m^2^/dose every 2 weeks; ?100 mg/dose/weekly.

Δ Age or plate count at diagnosis.

IVIG: intravenous immunoglobulin; anti-D: anti-D immunoglobulins; St: steroids; Cyc: cyclophosphamide; Az: azathioprine; VCR: vincristine; CSA: cyclosporine; Sp: splenectomy; Dan: danazol.

HCQ: Hydroxychloroquine; MMF: mycophenolate mofetil.

ES: Evans syndrome; SLE: Systemic lupus erythematosus; ALPS: Autoimmune Lymphoproliferative Syndrome.

# Secondary ITP included 14 Evans syndrome, 1 Lupus-related ITP, and 4 other auto-immune diseases associated ITP.

### Rituximab Dose and Schedule

Doses of rituximab were reported in 16 studies (265 patients) (see table 2). 224 (84.5%) patients received intravenously rituximab as a dose of 375 mg/m^2^/week for 1∼6 doses. Among that, 175 (66.0%) patients received 4 doses; 22 (8.3%) patients received 1 dose; 10 (3.8%) patients received 6 doses; 7 (2.6%) patients received 2 doses; 6 (2.3%) patients received 3 doses; 4 (1.5%) patients received 5 doses. 4 (1.5%) patients received a second course of 3∼4 doses of 375 mg/m^2^/week rituximab [22], [24], and 9 (3.4%) patients received dose escalation to 750 mg/m^2^/dose as no response after 3 doses [20]. 18 (6.9%) patients received 100 mg/dose/week for 4 doses [26], [27] considering the high cost of rituximab. 2 patients received 500 mg/m^2^/dose every 2 weeks for 2 doses [22]. 21 patients received 1 dose of 375 mg/m^2^ or 4 dose of 100 mg/dose/week, but without further dose information as published in abstract only [33].

### Platelet Count Response

#### Primary ITP

**Figure 2:**
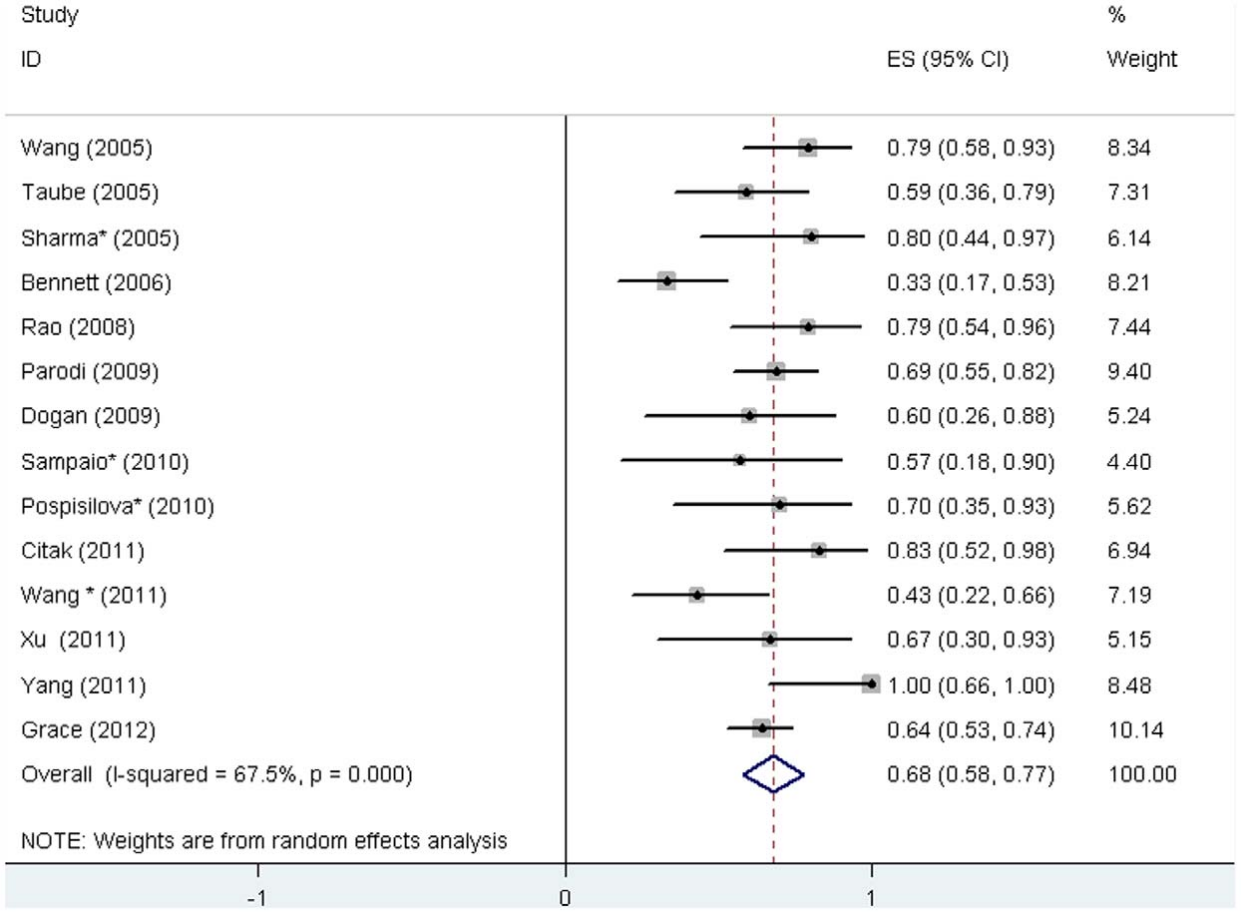
Response rate to rituximab in children with ITP. This forest plot is created by the software of STATA 11.1. Solid boxes indicate the response rate in each study. Horizontal lines indicated 95% CIs. The diamond indicates the pooled response rate (68%). Test of heterogeneity: I^2^ = 67.5%, P<0.001.

**Figure 3:**
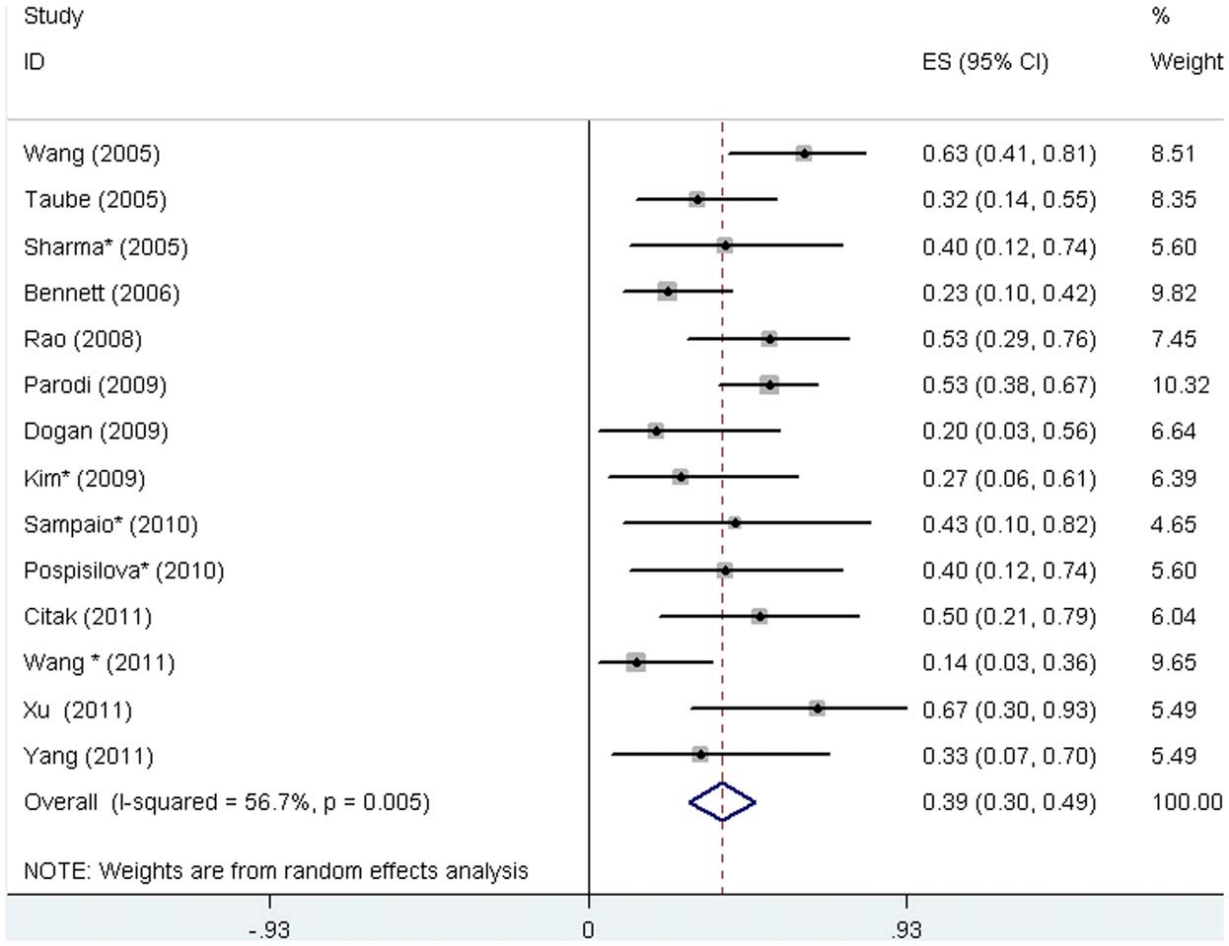
Complete response rate to rituximab in children with ITP. The diamond indicates the pooled complete response rate (39%). Test of heterogeneity: I^2^ = 56.7%, P = 0.005.

Response and complete response were defined with varied platelet count thresholds in the primary studies. We calculated the pooled response rate according to criteria defined in this systematic review. Response (platelet count ≥30×10^9^/L) after rituximab treatment was reported in 14 studies [16]–[18], [20], [21], [23], [25]–[29], [31]–[33] involving 312 patients, and was achieved in 201 patients (64.4%). 19 (6%) patients, although diagnosed as secondary ITP, were still included in the analysis as their data can not be separated. The response rate reported in the primary studies ranged from 33% [18] to 100% [27], and the pooled rate was 68% (95% CI, 58% to 77%). The heterogeneity between studies was statistically significant (P<0.001) (Figure 2). Complete response was reported in 14 studies [16]–[18], [20], [21], [23], [25]–[27], [29]–[33] involving 243 patients, and was achieved in 99 patients (40.7%). The reported complete response rate ranged from 14% [33] to 67% [26], and the pooled rate was 39% (95%CI, 30% to 49%). The heterogeneity was statistically significant (P = 0.005) (Figure 3).

#### Secondary ITP

4 studies [18], [19], [22], [24] reported data of 29 children with secondary ITP separately. 17 children with Evans syndrome associated ITP were included in 2 studies [18], [19], among that 11 (64.7%) children achieved response and 9 (52.9%) achieved complete response. 6 children with systemic lupus erythematosus and 6 patients with autoimmune lymphoproliferative syndrome associated ITP all (100%) achieved complete response. [22], [24].

### Time to Response and Response Duration

#### Primary ITP

Time to response was reported in 8 studies [17], [18], [21], [23], [25], [26], [27], [30] (97 patients with response), ranging from 0.3 to 17.0 weeks [23]. Individual level data was reported in 2 studies [21], [23] (40 patients with response), the combined median time to response was 3.0 (IQR, 1.0 to 3.6) weeks. Response duration was reported in 8 studies [17], [18], [21], [23], [25], [26], [27], [32] (97 patients with response), ranging from 0.9 to 74.8 months [23], but among that, 63 patients (64.9%) were still having ongoing response when the follow-up ended. Individual level data was reported in 4 studies [17], [21], [23], [32] (62 patients with response), the median response duration was 12.8 (IQR, 4.5 to 25.1) months, among that, 35 patients (56.5%) were having ongoing response when the follow-up ended.

#### Secondary ITP

7 patients with Evans syndrome associated ITP achieved response with time to response from 0.8 to 8 weeks, and none relapsed after 8 to 20 months follow-up [19]. 6 patients with systemic lupus erythematosus associated ITP achieved response with time to response from 1 to 12 weeks, among that, 2 patients relapsed at 12 and 17 months, but both achieved complete response again after a second course of rituximab, other patients had ongoing response from 6 to 22 months [22]. 2 of 6 patients with autoimmune lymphoproliferative syndrome associated ITP relapsed at 15 and 18 months, the other 4 patients had ongoing response from 5 to 36 months, but data of time to response was not reported [24].

### Predictors of Response to Rituximab

4 studies [18], [23], [28], [29] (175 patients) analyzed clinical characteristics associated with platelet count response to rituximab, however, their results varied. Bennett 2005 [18] (36 patients) found that attainment of response was weakly associated with diagnosis of Evans syndrome (P = 0.06), female sex (P = 0.14) and black race (P = 0.09), but the reported P value did not show significance association (P>0.05). Parodi 2009 [23] (49 patients) founded that median duration of ITP was significantly shorter in responders than in non-responders (P = 0.01). Grace 2012 [28], which was the largest (80 patients), showed that secondary ITP and response to steroid were strong predictors of response to rituximab, and was both suggested by result of univariate and multivariable analysis. Sharma 2005 [29] reported that patients showing a higher degree of response continued to be in remission for a longer period compared to ones with lesser degree of response.

### Toxicities

23 studies reported 108 adverse events in 91 patients. In 11 studies (190 patients) enrolling more than 5 patients, 78 (41.1%) patients experienced adverse events. Adverse events were classified from Grade 1 to 5 according to Common Terminology Criteria for Adverse Events (CTCAE) [46], and were described in table 3. In all reported 108 adverse events, 91 (84.3%) were mild to moderate, and the most frequently described adverse events are mild allergic reactions, including pruritus, urticaria, chills and fever. 7 patients developed serum sickness after 1 or 2 doses of rituximab, presented with fever, rash, arthralgia and fatigue, and in which, 3 cases were assigned to Grade 3–4, as 1 patient has an acute reaction followed by persistent arthralgia and 2 patients discontinued rituximab for serum sickness [17], [18]. 2 patients experienced immediate hypersensitivity reaction during rituximab infusion, which caused termination of the treatment [20], [45]. 4 patients developed infections that may be associated with rituximab, including 2 patients with varicella [18], [34], 1 patient with pneumonia [19], and 1 patient with life-threatening enteroviral meningoencephalitis presented with a progressive alteration in cognitive functions associated with aphasia and sensorimotor deafness [36]. 1 patient developed common variable immunodeficiency which caused prolonged hypogammaglobulinemia and increased susceptibility to infections [41]. 1 patient developed headache with white matter changes on brain MRI, as only described in abstract, no further information was reported [38]. No death associated with rituximab was reported.

**Table 3:** Adverse events observed after rituximab infusion in children with ITP.

Study	ITP Patients, n	Patients experienced adverse events, n	Adverse events (n)		
			Grade 1–2	Grade 3–4	Grade 5
Lorenzana, (2002)* [34]	3	1	NR	Viral infection (varicella) (1); Centralvenous line infection (1)	None
Bengtson, (2003) [35]	1	1	Rash(1); Elevated serum IgE level (1)	None	None
Quartier (2003) [36]	1	1	NR	Enteroviral meningoencephalitis (1)	None
Pusiol (2004) [37]	2	1	Fever and chill (1)	None	None
Sharma (2005)* [29]	10	3	Urticaria (1); Fever(2)	None	None
Wang, (2005) [17]	24	9	Pruritus (1); Throat tightness (1);Urticaria(3); Headache (3); Chest pain (1);Serum sickness (2); Low ANC (3)	Serum sickness (1)	None
Bennett, (2006) [18]	36	23	Chills, fever, and respiratorysymptoms (17)	Serum sickness (2); Viral infection(varicella) (1); Hypotension (1)	None
Yadav, (2006)* [38]	3	1	Drowsiness (1)	Hypotension(1); White matterchanges on MRI (1)	None
Bader-Meunier(2007) [19]	11	7	Vomiting(1); Facial edema(1);Urticarial rash(1)	Transient neutropenia (3); Pneumonia(1)	None
Bisogno (2007) [39]	1	1	Persistent B-cell depletion andhypogammaglobulinemia (1)	NR	None
Kim (2007) [40]	1	1	Fatigue and malaise (1)	NR	None
Gentner,(2008) [41]	1	1	NR	Common variable immunodeficiency (1)	None
Parodi, (2009) [23]	49	9	Urticaria (4); Mild headache (3);Chills (3); Fever (2)	None	None
Rao,(2008) [20]	19	15	Cough (4); Rash (3); Muscle cramps (3);Headache (2); Shortness of breath (1);Hypertension (1); Chills (1); Fever (1);Angioedema (1)	Immediate hypersensitivity reaction(1)	None
Adeli,(2009) [42]	2	2	Persistent hypogammaglobulinemia (2)	NR	None
Cooper (2009) [43]	1	1	Persistent B-celldepletion andhypogammaglobulinemia (1)	NR	None
Dogan, (2009) [21]	10	3	Itching and scraps(3)	None	None
Goto (2009) [44]	1	1	Serum sickness (1)	NR	None
Rao (2009) [24]	6	3	Persistent hypogammaglobulinemia (3)	NR	None
Sampaio (2010)* [32]	7	3	Pruritus (1); Urticaria (1); Vomiting (1)	None	None
Romanos-Sirakis (2011) [45]	1	1	Serum sickness (1)	Immediate hypersensitivity reaction(1)	None
Xu (2011) [26]	9	2	Fever and chill (2); Urticaria (2)	None	None
Yang (2011) [27]	9	1	Pruritus (1)	None	None

NR: not reported * Abstract.

Grade 1-Grade 2: Mild to moderate; Grade 3: Severe but not immediately life-threatening; Grade 4: Life-threatening; Grade 5: Death.

ANC: absolute neutrophil count; MRI: magnetic resonance imaging.

### Publication Bias

**Figure 4:**
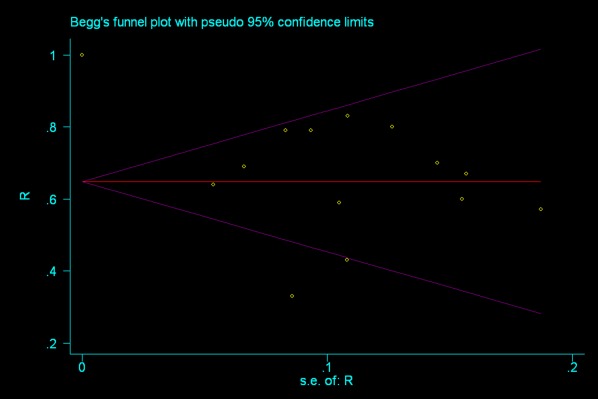
Begg funnel plot with pseudo 95% for studies reporting response. This plot is created by STATA 11.1. For each study, the response rate is plotted against its standard error. The funnel plot suggests no significant asymmetry, indicating no evidence of substantial publication bias.

The Egger and Begg test were conducted to investigate publication bias in 14 studies reporting response in patients with primary ITP. The Egger test indicated no evidence of publication bias (P = 0.987). Begg’s funnel plot with pseudo 95% (figure 4) was graphed in the logic that studies with smaller sample size have larger random error, thus are more dispersed, and the plot of effect estimates against standard errors would be skewed and asymmetrical in the presence of publication bias. In this review, the funnel plot suggests no significant asymmetry, indicating no evidence of substantial publication bias (P = 0.583).

## Discussion

This is the first systematic review that summarizes the efficacy and safety of rituximab in children with ITP, and this review is very important at this time as rituximab has been widely used off-label for children with ITP. The treatment of rituximab achieved response in 68% and complete response in 39% of patients with primary ITP. According to studies reporting individual data, the median time to response was 3.0 weeks and the median response duration was 12.8 months. Studies with a small number of patients with secondary ITP reported that response rate to rituximab was from 64.7%∼100%. Most adverse events were mild to moderate and no death was reported.

The response and complete response rate in children with primary ITP obtained in this review was 68% and 39%, respectively. It was similar to that reported in the previous systematic review on adults, which reported that overall response (platelet count >50×10^9^/L) and complete response (platelet count >150×10^9/^L) rate was 63% and 46% respectively [9]. Although response criteria differ in these two reviews, it may suggest that rituximab results in a similar response in adults and children with primary ITP.

The frequently administrated dose of rituximab is 375 mg/m^2^ per week for 4 weeks for both children and adult with ITP [9], although this dose was developed and approved for the treatment of lymphoma [47]. 1 study investigated the efficacy of a single dose of rituximab (375 mg/m^2^) in children, and reported a similar response and complete response rate as 4 doses [16]. 2 studies investigated a low dose rituximab of 100 mg/dose/week for 4 weeks in 18 children with primary ITP, which suggested that the lower dose of rituximab still reached a good response [26], [27]. Treatment of rituximab is still costly in the present time, and thus sometimes 4 doses of 375 mg/m^2^ are not affordable, especially for patients with poor accessibility to health resources. In addition, high dose and multiple dosing may increase risk of adverse events. Low dose of rituximab may be promising for patients with ITP, and more studies are needed to investigate that.

Most of adverse events associated with rituximab in children with ITP were mild to moderate infusional reactions. More severe adverse effects included serum sickness, common variable immunodeficiency, severe virus infection including enteroviral meningoencephalitis, and white matter changes. 2 children developed viral infection after rituximab treatment, but they were both previously treated with other immunosuppressive agents like steroids, vincristine, and cyclosporine A, it is difficult to establish a direct association with rituximab [18], [34]. In addition, a systematic review in cancer patients did not find any increase of infection caused by monoclonal antibodies [48]. The case of common variable immunodeficiency in patients with ITP after rituximab treatment was reported in an 8-year-old child [41], however, it was not clear whether it was caused by rituximab, as ITP might be the first clinical manifestation before development of common variable immunodeficiency. 1 patient developed headache and MRI showing white matter changes [38], but without detailed information as published only in abstract, it is not clear whether the children developed progressive multifocal leukoencephalopathy, although it is reported that there was a potential of progressive multifocal leukoencephalopathy among rituximab-treated patients [49]. No death was found in this review, but 9 cases of death (2.9%) were reported in studies on adults [9]. The mortality reported in adults treated with rituximab might be overestimated and these cases might be explained by long courses of complex treatment regimens or the selection of patients with advanced disease.

All the studies eligible for efficacy analysis in this review failed to compare effect between rituximab with non-rituximab group. The response rate obtained may be influenced by many potential factors, such as treatments before and combined with rituximab, cause and development of ITP. Studies on this topic with better methodological design like randomized controlled studies are urgently needed. Consecutive enrolling of patients can help to reduce selection bias in prospective case series studies, unfortunately, few studies provided information on that, and this needs improvement in future studies.

## Supporting Information

Table S1 Search strategy for PUBMED, EMBASE and CENTRAL.
(DOC)

Table S2 PRISMA checklist.
(DOC)
