# Supplementary material for: Rituximab for Children with Immune Thrombocytopenia: A Systematic Review
Source: PLoS One. 2012 May 30;7(5):e36698. doi: 10.1371/journal.pone.0036698 (PMC3364261; doi:10.1371/journal.pone.0036698)
Supplement: Table S1 — Search strategy for PUBMED, EMBASE and CENTRAL. (DOC) [file pone.0036698.s001.doc]

**Table S1 Search Strategy For PUBMED, EMBASE And CENTRAL**

| **Data base** | **Search strategy** |
| --- | --- |
| PUBMED | #1 Rituximab [Supplementary Concept]  #2 Ritux*  #3 Mabthera*  #4 "Purpura, Thrombocytopenic, Idiopathic"[MESH]  #5 Thrombocytopeni*  #6 ITP  #7 Child*  #8 Toddler*  #9 Infant*  #10 Adolescen*  #11 Pediatr*  #12 Paediatr*  #13 #1 OR #2 OR #3  #14 #4 OR #5 OR #6  #15 #7 OR #8 OR #9 OR #10 OR #11 OR #12  #16 # 13 AND #14 AND #15 |
| EMBASE | #1 Rituximab[emtree]  #2 Ritux*  #3 Mabthera*  #4 “idiopathic thrombocytopenic purpura”[emtree]  #5 Thrombocytopeni*  # 6 ITP  #7 Child*  #8 Toddler*  # 9 Infant*  #10 Adolescen*  #11 Pediatr*  # 12Paediatr*  #13 #1 OR #2 OR #3  #14 #4 OR #5 OR #6  #15 #7 OR #8 OR #9 OR #10 OR #11 OR #12  #16 #13 AND #14 AND #15 |
| CENTRAL | #1 Ritux*  #2 Mabthera*  #3 idiopathic thrombocytopenic purpura'[MESH]  #4 Thrombocytopeni*  #5 ITP  #6 Child*  #7 Toddler*  # 8 Infant*  #9 Adolescen*  #10 Pediatr*  #11 Paediatr*  #12 #1 OR #2  #13 #3 OR #4 OR #5  #14 #6 OR #7 OR #8 OR #9 OR#10 OR #11  #15 #12 AND #13 AND #14 |
